# SiamQuality: a ConvNet-based foundation model for photoplethysmography signals

**DOI:** 10.1088/1361-6579/ad6747

**Published:** 2024-08-12

**Authors:** Cheng Ding, Zhicheng Guo, Zhaoliang Chen, Randall J Lee, Cynthia Rudin, Xiao Hu

**Affiliations:** 1 Department of Biomedical Engineering, Georgia Institute of Technology, Atlanta, GA, United States of America; 2 Department of Electrical and Computer Engineering, Duke University, Durham, NC, United States of America; 3 Department of Computer Science, Emory University, Atlanta, GA, United States of America; 4 School of Medicine, University of California at San Francisco, San Francisco, CA, United States of America; 5 Department of Computer Science, Duke University, Durham, NC, United States of America; 6 Nell Hodgson Woodruff School of Nursing, Emory University, Atlanta, GA, United States of America; 7 Department of Biomedical Informatics, Emory University School of Medicine, Atlanta, GA, United States of America

**Keywords:** foundation model, physiological data, PPG signal quality

## Abstract

*Objective*. Physiological data are often low quality and thereby compromises the effectiveness of related health monitoring. The primary goal of this study is to develop a robust foundation model that can effectively handle low-quality issue in physiological data. *Approach*. We introduce SiamQuality, a self-supervised learning approach using convolutional neural networks (CNNs) as the backbone. SiamQuality learns to generate similar representations for both high and low quality photoplethysmography (PPG) signals that originate from similar physiological states. We leveraged a substantial dataset of PPG signals from hospitalized intensive care patients, comprised of over 36 million 30 s PPG pairs. *Main results*. After pre-training the SiamQuality model, it was fine-tuned and tested on six PPG downstream tasks focusing on cardiovascular monitoring. Notably, in tasks such as respiratory rate estimation and atrial fibrillation detection, the model’s performance exceeded the state-of-the-art by 75% and 5%, respectively. The results highlight the effectiveness of our model across all evaluated tasks, demonstrating significant improvements, especially in applications for heart monitoring on wearable devices. *Significance*. This study underscores the potential of CNNs as a robust backbone for foundation models tailored to physiological data, emphasizing their capability to maintain performance despite variations in data quality. The success of the SiamQuality model in handling real-world, variable-quality data opens new avenues for the development of more reliable and efficient healthcare monitoring technologies.

## 1. Introduction

Foundation models, particularly those with transformer architectures as the backbone (Vaswani 2017), have significantly influenced the landscape of artificial intelligence in recent years. Their remarkable ability to capture and generate human language, as well as to process and interpret complex visual information, has set new standards in language and language-vision tasks (Min 2021, Du *et al* 2022, Chen *et al* 2023, Zhao 2023). The potential reward is high if such successes could be replicated in other domains. For example, as highlighted in the editorial by Hu (2024), there is significant potential for developing foundation models for physiological data using self-supervised learning techniques. These models could be leveraged to analyze intricate physiological signals and facilitate a variety of health monitoring tasks.

One reason that physiological measurement tasks are different than language or general computer vision tasks is that physiological data is of notoriously low quality (Clifford *et al* 2012), making it difficult to train and deploy efficient models. The reliability of physiological signals is often compromised by the presence of artifacts and noise, which can stem from patient movement, sensor displacement, and physiological variations. These artifacts can significantly distort the signal, leading to inaccurate assessments in clinical diagnostics and remote health monitoring. The inherent noise, incompleteness, and inconsistency, compounded by the complexities of human physiology and the unpredictable conditions of free-living data collection environments (Charlton 2023), necessitate robust model architectures to achieve reliable results.

In this study, we propose SiamQuality, a new foundation model to address the issue of physiological data quality. Instead of using transformers, which are complex and difficult to troubleshoot, we utilize CNNs as the backbone, given their natural ability to represent sequences (Zhao *et al* 2017, Liu *et al* 2018, Tang *et al* 2020). To train and test our approach, we leverage a large dataset of photoplethysmography (PPG) signals collected from hospitalized intensive care patients.

**Figure 1.**
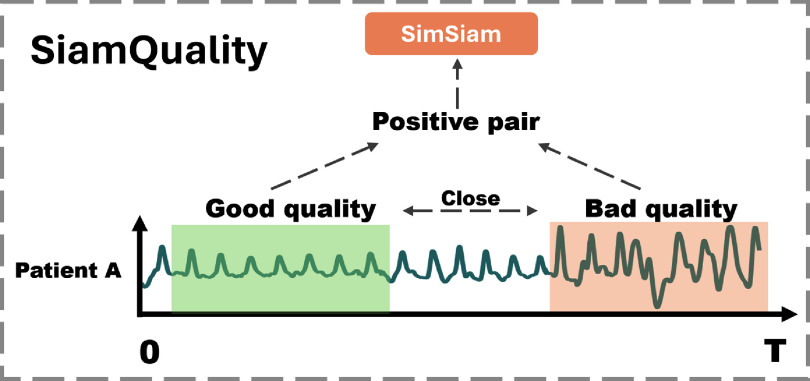
SiamQuality handles variations in data quality by ensuring that low and high quality signals from similar physiological states map to similar feature representations.

As shown in figure 1, the proposed SiamQuality is based on the SimSiam architecture (Chen and He 2021), with a novel signal quality-based pairing mechanism. This methodology leverages the power of contrastive learning to extract robust features from PPG signals, alleviating the influence of low signal quality. The rationale behind this approach is that representations of PPG signals from similar physiological states, as learned by CNNs, should be similar; a low quality PPG signal in a temporal vicinity of a high quality one from the same person is likely to be from a similar physiological state.

As a foundation model, SiamQuality meets three criteria: (i) It is pre-trained on large-scale data. In this study, we collected over 36 million 30 s PPG pairs (600 000+ h) from 21 000 patients as the training data for SiamQuality. (ii) It accommodates increasing complexity. We use a CNN—specifically ResNet (He *et al* 2016)—as the backbone and successfully observe a performance improvement of SiamQuality when increasing model size. (iii) It is adaptable to a variety of tasks. We conducted extensive experiments, fine-tuning SiamQuality on six different PPG-based downstream classification and regression tasks. Experimental results validate that it improves performance on all downstream tasks, achieving or exceeding state-of-the-art (SOTA) performance. Notably, on the tasks of respiratory rate estimation and atrial fibrillation detection, it outperforms state-of-the-art models by 75% and 5%, respectively.

This study goes beyond merely presenting an efficient foundation model for PPG signals, it provides an alternative solution that emphasizes the often-overlooked aspect of data quality in the development of foundation models.

## 2. Related work

**Foundation models for physiological data**. Foundation models have now been built in many areas, including language and computer vision, though only a few of them focus on physiological data. HeartBeit (Vaid 2023) utilizes masked image modeling to develop a transformer model based on vision for analyzing electrocardiogram (ECG) waveforms. This pre-training method improves the model’s accuracy and provides a degree of explainability for its predictions, as demonstrated by the results of GRAD-CAM analysis. Another foundation model, the Biosignal Transformer Model (BIOT) (Yang *et al* 2023), learns embeddings for biosignals, mainly electroencephalogram (EEG) signals. BIOT enables effective knowledge transfer across different datasets and allows joint training on multiple sources. In Li *et al* (2023), the authors enhance the ECG representation learning by fine-tuning the latent space generated from a frozen large language model with text from ECG reports. Apple also developed foundation models for wearable biosignals (Abbaspourazad *et al* 2023), specifically targeting PPG and ECG. These models were tested for their ability to predict various participant demographics and health conditions, including age, body mass index (BMI), and biological sex. The results revealed that these pre-trained foundation models exhibited high accuracy in these predictions.

**Contrastive learning for time series data**. Positive and negative pairs are often needed in self-supervised contrastive learning. For instance, TS2Vec (Yue 2022) adopts a hierarchical contrastive learning framework to handle time series augmentations, where each time step is represented as a point in an embedding space. CoST (Woo *et al* 2022) encodes disentangled trends and seasonal representations through contrastive learning. The above-mentioned studies require negative pairs for the self-contrastive learning, where a negative pair consists of parts from two different time series. However, the authors of SimTS (Zheng *et al* 2023) have conducted experiments showing that current ways of constructing negative pairs are not efficient for time series data, meaning that the performance of the model does not benefit from the negative pairs. Instead, they propose that two consecutive time series can be combined as one positive pair, where positive pairs are pulled together in latent space. With SimSiam, which only requires positive pairs, SimTS outperformed TS2Vec (Yue 2022) and CoST (Woo *et al* 2022) in experiments of Zheng *et al* (2023). In our study, we also use the SimSiam architecture and have included a modified SimTS (Zheng *et al* 2023) as one of the baselines, discussed in section 3.5.1.

### 2.1. Method

### 2.2. Preliminaries and problem formulation

Our dataset $K = \{x_i,y_i,t_i\}_{i = 1}^N$ consists of PPG strips *x_i_*, each associated with a label *y_i_* representing the percentage of artifacts (perturbations in the signal) in *x_i_* (ranging from 0% to 100%), where *t_i_* is the collection time. Our goal is to learn a robust representation that is minimally affected by artifacts.

### 2.3. Signal quality pairing

**Figure 2.**
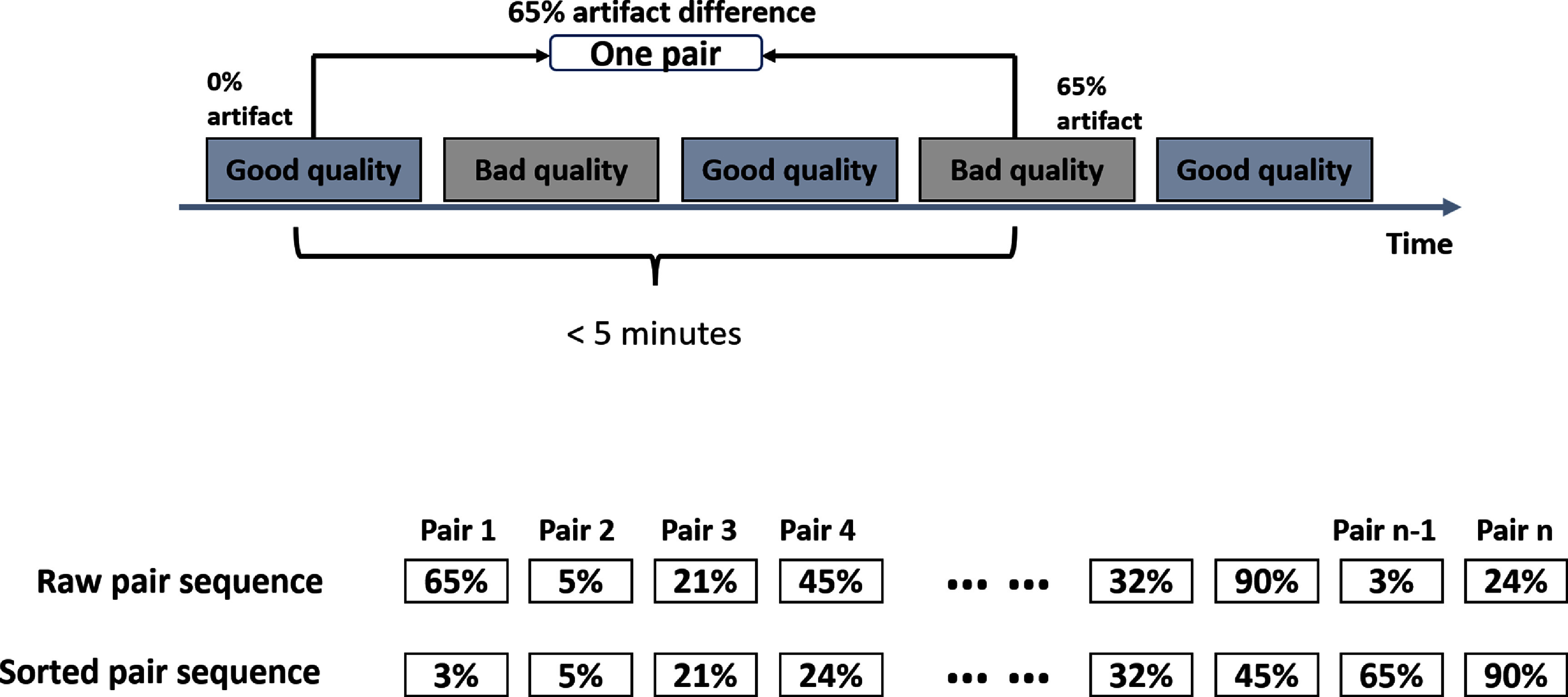
The mechanism for signal quality pairing.

We used continuous PPG recordings collected from adult intensive care units (ICUs) to generate pairs of varying quality, as illustrated in figure 2. To categorize each 30 s PPG segment’s quality, we implemented a quality assessment method previously developed in (Chen *et al* 2023). This method classifies each segment into a spectrum of quality from high to low, based on the percentage of artifacts present. For *x_i_*, the collection time *t_i_* for each strip is also recorded. In our methodology, segments identified as high quality ($y_i = 0$) serve as anchor points for beginning a search for a nearby low-quality signal.

As reported in algorithm 1, from each anchor point, we search for segments within a 5 min window that is low quality (having high *y_i_*). Among these, we select the segment with the highest temporal distance from the anchor, ensuring a varied degree of comparison in terms of artifacts and time. This approach allows us to construct pairs of PPG segments that are physiologically similar but differ significantly in their quality, as dictated by the presence of artifacts. These pairs are then used to train our model, enabling it to learn robust representations that are minimally affected by artifacts.

**Table:** 

**Algorithm 1.** SiamQuality with curriculum learning.
1: **Input:** Dataset $ K = \{x_i, y_i, t_i\}^N_{i = 1} $ of PPG signals *x_i_* with artifact percentage *y_i_* and collection time *t_i_*
2: **Output:** Specify the output if needed
3: **Step 1:** Initialization
4: Initialize Encoder *E*, Projector *P*, Predictor *D*, and Classifier
5: Define cosine similarity loss function $ L_{\mathrm{cosine}} $
6: **Step 2:** Data Preparation
7: **for** **each** PPG signal *i* such that $ (x_i, y_i = 0) $ **do**
8: Find PPG *x_j_* that
$j = \max\left\{k | \left(|t_k - t_i| < 5\ \text{mins}\right) \textrm{AND } \left(y_k > 0.2\right)\right\}$
9: Pair PPG signals $ (x_i, x_j, c_i) $ where $ c = y_j - y_i $
10: **end for**
11: Sort the training data (X, Y, C) based on C.
12: **Step 3:** Curriculum Learning and Model Training
13: **for** **each** pair $ (x_{\mathrm{good}}, x_{\mathrm{bad}}) $ **do**
14: Encode signals: $ h_{\mathrm{good}} = E(x_{\mathrm{good}}), h_{\mathrm{bad}} = E(x_{\mathrm{bad}}) $
15: Project encoded signals: $ z_{\mathrm{good}} = P(h_{\mathrm{good}}), z_{\mathrm{bad}} = P(h_{\mathrm{bad}}) $
16: Predictor: $ p_{\mathrm{good}} = D(z_{\mathrm{good}}), p_{\mathrm{bad}} = D(z_{\mathrm{bad}}) $
17: Compute contrastive loss: $ L = \frac{1}{2} (L_{\mathrm{cosine}}(p_{\mathrm{good}}, z_{\mathrm{bad}}) + L_{\mathrm{cosine}}(p_{\mathrm{bad}}, z_{\mathrm{good}})) $
18: Update model parameters to minimize *L*
19: **end for**

In addition, we implemented curriculum learning (Wang *et al* 2021) by systematically feeding the model training pairs, starting with small artifacts, and later introducing larger artifacts. The size of the artifact between a clean and not-clean signal is quantified by a measure $C(x_1,x_2 ) = |y_1- y_2 |$. After sorting the training data based on *C*, the model is initially trained with pairs having lower $C(x_1,x_2)$ and progressively introduced to pairs with higher differences.

### 2.4. Contrastive learning framework using simsiam

**Figure 3.**
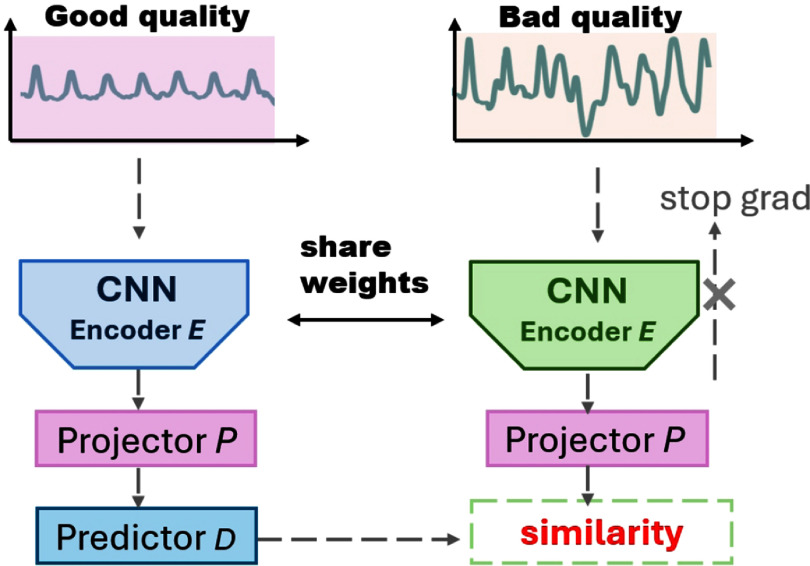
SiamQuality: SimSiam architecture with quality pairing augmentation.

Our approach builds from the SimSiam architecture (Chen and He 2021), adapted for PPG signal processing, as shown in figure 3. The key components of our model include an encoder *E*, a projector *P*, and a predictor *D*.

#### 2.4.1. Encoder and projector

The encoder *E* transforms the input signal *x* into a representation $h = E(x)$. The projector *P* then maps this representation into a lower-dimensional space, $z = P(h)$, suitable for contrastive learning. The encoder in our model utilizes a ResNet backbone. The encoder processes the input signal to extract and condense information into a high-level, compact feature representation. This representation captures the essential characteristics of the input data, crucial for the effectiveness of the subsequent layers (projector and predictor). Comprising three fully-connected (dense) layers, the projector further transforms the dense representation output by the ResNet encoder. This additional transformation prepares the data for a specific type of comparison or contrastive task. The projector maps the encoded features into a space where the model can more effectively optimize the contrastive loss function.

#### 2.4.2. Predictor

The predictor *D* maps the projected representation *z* to the space where contrastive loss is applied. For a pair of signals *x*_1_ (high quality) and *x*_2_ (low quality), their latent representations are $z_1 = P(E(x_1 ))$ and $z_2 = P(E(x_2 ))$, respectively. The predictor’s output for *x_i_* is $p_i = D(z_i)$. Similar to the projector, the predictor also consists of three fully-connected layers. Notably, the input and output dimensions of the predictor are the same, ensuring that the sizes of *p_i_* and *z_i_* are identical. However, its role differs in that it predicts properties from the transformed representations. In SiamQuality, the predictor is used to enforce consistency between differently views of the same data, thereby improving the learning of invariant and robust features.

#### 2.4.3. Contrastive loss function

The cosine similarity loss $L_{\mathrm{cosine}}$ is used to minimize the distance between the predicted latent feature vectors within the pair:

\begin{align*}L_{\mathrm{cosine}}\left(x_1, x_2\right) = -\frac{p_1\cdot z_2}{\|p_1\|_2\cdot \|z_2\|_2} - \frac{p_2\cdot z_1}{\|p_2\|_2\cdot \|z_1\|_2}\end{align*}

where · denotes the dot product, and $\|\cdot\|_2$ represents the *L*_2_ norm.

## 3. Experimental setup and results

**Figure 4.**
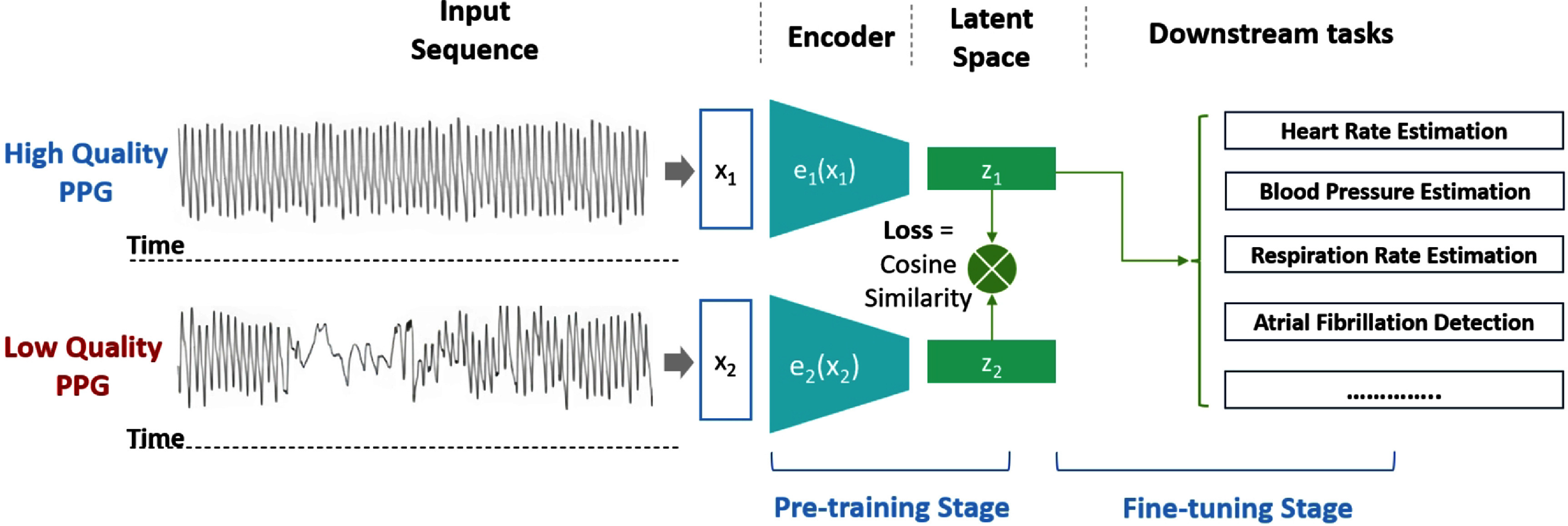
The workflow for SiamQuality: pre-training and fine-tuning.

As illustrated in figure 4, SiamQuality is set up to learn robust features from PPG signals, irrespective of their quality, by using a shared-weight encoder and a similarity-based loss function. After pre-training, the model can be fine-tuned to perform various clinical and physiological downstream tasks.

### 3.1. Data

The dataset used for model pre-training was obtained from routine patient monitoring systems in the intensive care units of the University of California, San Francisco (UCSF) Medical Center. This collection was conducted with an approved waiver of written patient consent under the UCSF Institutional Review Board (IRB number: 14-13262). The retrospective use of this fully de-identified data was conducted according to the terms of a data use agreement between UCSF and Emory University.

The waveform dataset, which includes physiological signals, cardiac arrhythmia alarms, and linked electronic health records (EHR) from over 24 100 patients, was collected at the UCSF Medical Center between March 2013 and December 2018. The demographics of the patients in this dataset are reported in table 1. In the waveform dataset, a total of 2.6 million hours of continuous signals were collected, including seven-lead ECG (I, II, III, V, AVR, AVL, AVF), one-channel PPG signal, and one-channel respiratory rate. All signals are sampled at 240 Hz.

**Table 1.** Characteristics of Participants Enrolled in UCSF medical center.

Characteristics	Total Cohort (*N* = 28 539)
Sex	
Female	13 203 (46.2%)
Male	15 330 (53.7%)
Others	6 (0%)
Age	
$ \unicode{x2A7E} $65 yr	12 157 (42.6%)
55–64 yr	5370 (18.8%)
40–54 yr	4372 (25.3%)
22–39 yr	2715 (9.5%)
$ < $22 yr	3925 (13.8%)
Race or ethnic group	
White or Caucasian	15 890 (55.7%)
Black or African American	2159 (7.4%)
Asian	4364 (15%)
Unknown/Declined	1149 (4%)
Others	4913 (16.9%)
Native Hawaiian	426 (1.46%)
American Indian	212 (0.7%)

### 3.2. Data preprocessing

Continuous PPG recordings were divided into discrete, 30 s segments without overlap. Each of these segments was first downsampled to a frequency of 40 Hz, followed by min-max normalization to standardize the signal range. To evaluate the quality of these signals, a previously developed binary PPG signal quality assessment tool (Pereira *et al* 2019) was employed, categorizing each segment as either ‘high quality’ or ‘low quality.’ For segments classified as having low quality, an additional analysis was applied using another signal quality segmentation model (Chen *et al* 2023). This model is specifically designed to segment the locations of artifacts within each 30 s PPG signal, therefore it can be used to quantify the percentage of artifacts present in each low quality signal.

### 3.3. Downstream tasks

As illustrated in figure 4, after the pre-training stage, we selected a range of diverse public downstream tasks to test the effectiveness of our method, allowing for reproducibility and validation of our results by the broader research community. Table 2 provides an overview of the datasets and their respective statistics.

**Table 2.** Statistics of public datasets for various downstream tasks.

Task	No. Subjects
Heart Rate Estimation	
Signal Type: ECG, PPG, Accelerometer	
TROIKA (Zhang *et al* 2014)	12
Dalia (Reiss *et al* 2019)	15
WESAD (Schmidt *et al* 2018)	17
Blood Pressure Estimation	
Signal Type: ECG, PPG, ABP Waveforms	
PulseDB (MIMIC-III+VitalDB) (Wang *et al* 2023a)	5361
Respiratory Rate Estimation	
Signal Type: PPG	
BIDMC PPG and Respiratory Dataset (Pimentel *et al* 2016)	53
AF Detection	
Signal Type: PPG	
Stanford AF dataset (Torres-Soto and Ashley 2020)	148

### 3.4. Performance evaluation metrics

In our study, we employ distinct evaluation metrics tailored to the nature of the tasks. For regression tasks, the Mean Absolute Error (MAE) is used. For classification tasks, we use the F1 score. MAE measures the accuracy of a model in regression tasks. It calculates the average magnitude of the errors in a set of predictions:

\begin{align*}\text{MAE} = \frac{1}{n}\sum_{i = 1}^n \left|y_i-\hat{y}_i \right|.\end{align*}

F1 is defined by:

\begin{align*}\text{F1} = 2\times \frac{\text{Precision}\times \text{Recall}}{\text{Precision}+\text{Recall}}, \quad \text{Precision} = \frac{\text{TP}}{\text{TP}+\text{FP}}, \quad \text{Recall} = \frac{\text{TP}}{\text{TP}+\text{FN}}\end{align*}

where TP, FP, FN stands for true positive, false positive, and false negative, respectively.

**Figure 5.**
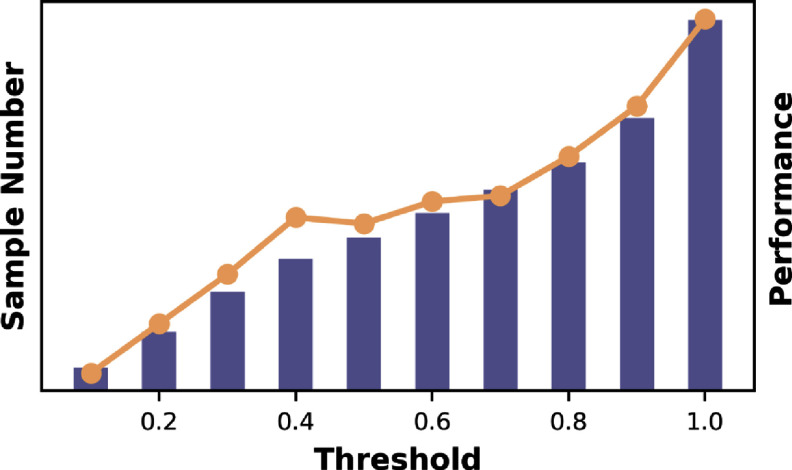
AT-curve. The horizontal axis represents the upper limit of the signal quality for each subgroup. The height of each bar denotes the sample size for each respective subgroup. The line plot shows MAE within each subgroup.

Additionally, we introduce a new metric, the Artifact Tolerance Curve (AT-Curve), illustrated in figure 5. The AT-Curve is designed to assess the robustness of our models across varying levels of signal quality within the test data. The level of signal quality is calculated by the method of Chen *et al* (2023), as introduced in section 2.3. The horizontal axis represents the upper limit for the signal quality of each subgroup, with values ranging from 0 to 1.0. A value of 1.0 represents the entire test set, while a value of 0 corresponds to the subset with the highest signal quality (i.e. the cleanest subset). The height of each bar on the AT-Curve denotes the sample size for each respective subgroup.

Overlaying the bars, a line plot traces the performance of our models-measured in terms of MAE for regression tasks and F1 score for classification tasks-within each of these subgroups. This line plot provides shows how our models’ performance varies with the quality of the PPG signals. The AT curve serves dual purposes: it illustrates the distribution of signal quality within our test data and maps our models’ performance against this distribution. The AT-curve thus offers a comprehensive view of our models’ efficacy across a spectrum of signal qualities, highlighting their robustness and adaptability in real-world scenarios.

### 3.5. Experimental results

#### 3.5.1. Comparison between different contrastive learning methods

In the first experiment, we conducted a comprehensive evaluation of several self-supervised learning (SSL) models: SimCLR (Chen *et al* 2020), SwAv (Zhu *et al* 2020), MOCO (He *et al* 2020), BYOL (Grill 2020), and SimSiam (Chen and He 2021). Methods in the top half of table 3 all used the same augmentation method, which is to randomly select from (Gaussian noise, powerline noise, and flipping) within each mini-batch. The original signal and its augmented counterpart are combined as one positive pair, and two different signals are combined as one negative pair. Among the five SSL approaches, only BYOL and SimSiam do not require negative pairs.

**Table 3.** Comparison between different contrastive learning methods.

Base Encoder: Resnet50	Augmentation Method	HR (MAE—beats/min)			RR (MAE—breaths/min)	AF (F1 Score)	BP (MAE—mmHg)
		TROIKA	Dalia	WESAD	BIDMC	Stanford	PulseDB
SimCLR (Chen *et al* 2020)		4.92 ± 2.56	7.53 ± 3.89	6.73 ± 1.88	2.34 ± 0.02	0.59	11.40 ± 5.34
SwAV (Zhu *et al* 2020)		4.82 ± 3.14	7.15 ± 2.84	6.52 ± 1.99	2.04 ± 0.06	0.64	11.22 ± 6.78
MoCo (He *et al* 2020)	Conventional	4.70 ± 2.78	7.28 ± 3.65	6.53 ± 2.67	2.17 ± 0.04	0.63	11.17 ± 7.15
BYOL (Grill 2020)	Augmentation	4.97 ± 2.97	7.50 ± 1.92	6.77 ± 3.14	2.41 ± 0.03	0.61	11.40 ± 5.89
SimSiam (Chen and He 2021)		4.71 ± 3.33	7.34 ± 3.29	6.65 ± 2.28	2.33 ± 0.03	0.63	10.71 ± 7.42
Modified SimTS (Zheng *et al* 2023)	Temporal Sampling	4.92 ± 2.34	7.31 ± 2.47	6.54 ± 2.44	2.08 ± 0.03	0.63	10.94 ± 6.03
SiamQuality (Ours)	+ Quality pairing	4.73 ± 3.02	7.15 ± 2.15	6.35 ± 1.67	1.96 ± 0.03	0.65	10.65 ± 5.57
SiamQuality (Ours)	+ Curriculum learning	**4.69 ± 2.89**	**7.02 ± 1.76**	**6.27 ± 1.73**	**1.84 ± 0.03**	**0.68**	**10.32 ± 7.69**

We now investigate different augmentation methods with SimSiam, as reported in the bottom half of table 3. In the original SimTS (Zheng *et al* 2023) approach, two consecutive time series were utilized. We modified this by using one signal and pairing it with a random 5 min neighboring signal, which we refer to as Modified SimTS. Then, we added consideration of signal quality into the temporal sampling (using the method described in section 2.3), and we further added curriculum learning as the third augmentation method.

The results show a trend of improvement with each successive augmentation strategy, for all datasets, for all of the tasks. SiamQuality yielded the best results, for all of the tasks (heart rate prediction, RR prediction, AF detection, and BP prediction).

#### 3.5.2. SimSiam with different model sizes

We investigated the performance of SimQuality as a function of model complexity, as reported in table 4. The results reveal a clear trend: as the complexity of the ResNet architecture (He *et al* 2016) increases, there is a marked improvement in performance. This trend is evident across all metrics, including MAE for Heart Rate, Respiratory Rate, and Blood Pressure, as well as Atrial Fibrillation detection. ResNet152, being the most complex model, consistently exhibited the best performance. However, ResNet152 is more expensive to train than ResNet50. The experiments in the remaining subsections do not require as much computation as earlier experiments, so we use ResNet152 from now on.

**Table 4.** SiamQuality with different model sizes.

Dataset	Resnet50	Resnet101	Resnet152
TROIKA (MAE)	4.69 ± 2.89	4.66 ± 3.13	**4.59 ± 2.93**
Dalia (MAE)	7.02 ± 1.76	6.92 ± 1.95	**6.80 ± 1.87**
WESAD (MAE)	6.27 ± 1.73	6.07 ± 1.89	**5.88 ± 2.34**
BIDMC (MAE)	1.84 ± 0.03	1.53 ± 0.01	**0.89 ± 0.01**
Stanford (F1)	0.68	0.69	**0.71**
PulseDB (MAE)	10.32 ± 7.69	9.64 ± 7.03	**8.60 ± 6.93**

#### 3.5.3. Comparing with previous work on each downstream task

We compared our model’s performance against the previous state-of-the-art (SOTA) results for each specific task. However, some of these studies did not provide uncertainty measures for their results, such as CurToss in the Troika dataset. Additionally, the Stanford AF dataset comes pre-split into training, validation, and test sets, allowing only for single-fold training; therefore, no uncertainty measures could be provided for this dataset.

**Figure 6.**
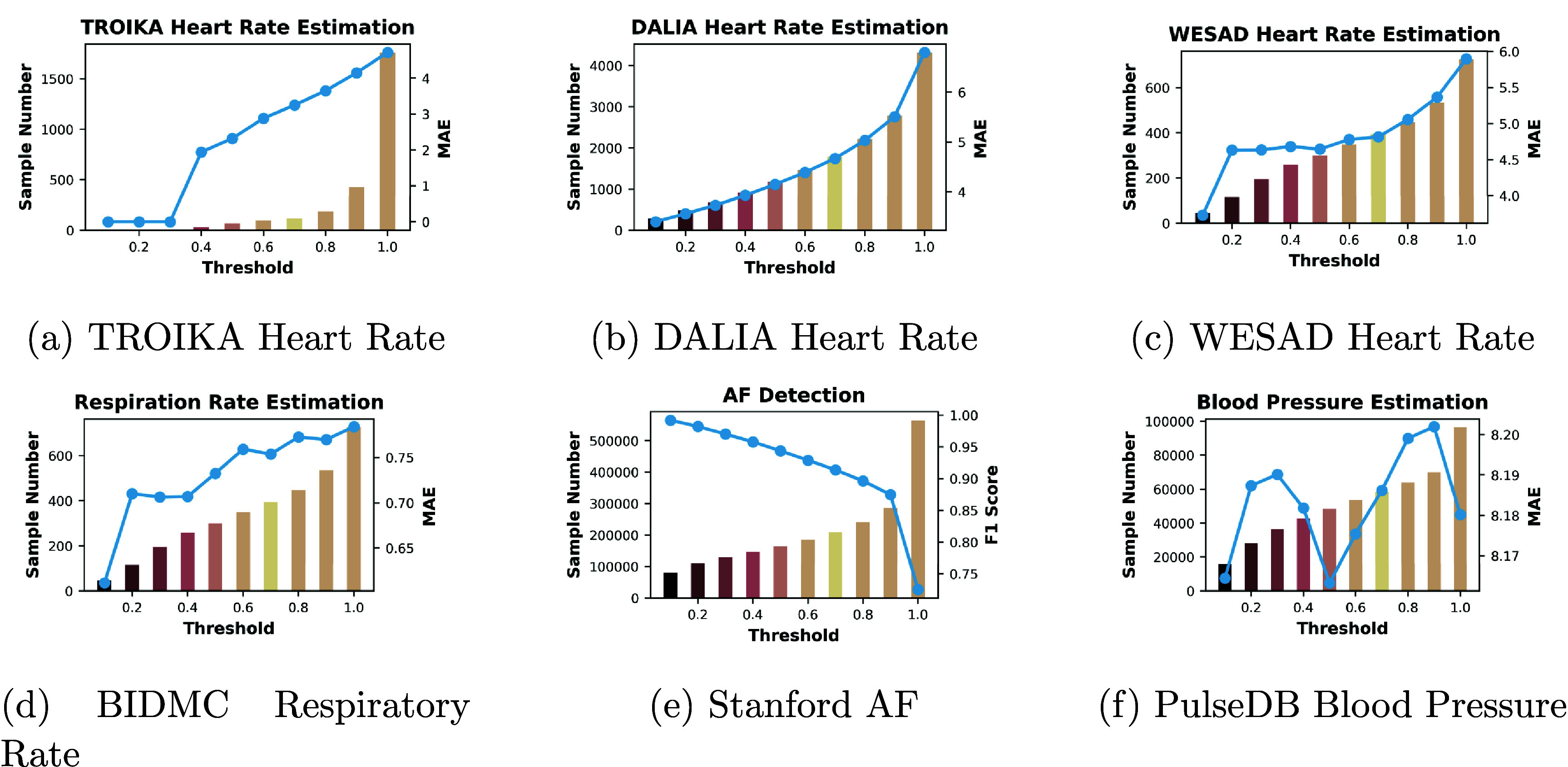
AT-Curve for all downstream tasks.

As reported in table 5, the studies we highlighted in gray show significantly better MAE values. This improvement is attributed to the additional step they introduced to exclude poor-quality signals before model training and testing. But note that it is important to state that low quality signals were omitted because MAE values are not comparable between cases when they are omitted from the test set and when they are not. Comparing DeepPPG to our method, which did not discard any signals, we find that we perform better on Dalia and WESAD but are slightly worse in TROIKA. Through the AT curve shown in figures 6(a)–(c), we can see that DALIA and WESAD have a more uniform distribution of artifact levels within each bin, while TROIKA displays a significant skew towards noisy signals, with a majority of signals exhibiting over 90% artifacts. In addition, from the AT-curve, we can observe that, in the case of DALIA, discarding signals containing over 60% of artifacts allows us to surpass the performance of BeliefPPG. For WESAD, the threshold to exceed BeliefPPG involves discarding signals with artifact levels above 20%. To outperform BeliefPPG in the TROIKA dataset, it is necessary to exclude signals with more than 40% artifacts.

**Table 5.** Test set results compared with SOTA in HR datasets (MAE). The rows in gray are incomparable to rows in black since they omit low quality signals from the evaluation.

Datasets	TROIKA	Dalia	WESAD
Deep PPG (Reiss *et al* 2019)	4.00 ± 5.4	7.65 ± 4.2	7.47 ± 3.3
PPGNet (Shyam *et al* 2019)	3.36 ± 4.1	—	—
CurToSS (Zhou and Selvaraj 2020)	2.20	5.00 ± 2.8	6.40 ± 1.8
TAPIR (Huang and Selvaraj 2020)	2.50 ± 1.2	4.60 ± 1.4	4.20 ± 1.4
BeliefPPG (Bieri *et al* 2023)	1.88 ± 0.7	4.26 ± 1.6	3.81 ± 1.9
Our methods	4.59 ± 2.93	6.80 ± 1.87	5.88 ± 2.34

A range of methods are compared for RR prediction, as reported in table 6. Compared to the previous SOTA methods, the LSTM model demonstrated the strongest performance with an MAE of 1.51. However, our method outperformed all these techniques, achieving the lowest MAE of 0.89, indicating its superior accuracy in estimating respiratory rate. The AT curve presented in figure 5(d) shows that performance does not uniformly improve with better signal quality.

**Table 6.** Test set results compared with SOTA in Respiratory Rate dataset. Uncertainty was not reported in the first four methods’ papers.

Methods	RR (MAE)
Multiple auto-regression (Pimentel *et al* 2016)	4.0
EEMD + PCA (Nilsson *et al* 2000)	3.5
EEMD + Kalman filter (Motin *et al* 2017)	1.9
Smart fusion (Karlen *et al* 2013)	5.8
LSTM (Kumar *et al* 2022)	1.51 ± 0.03
LSTM+Attn (Kumar *et al* 2022)	1.56 ± 0.03
Our methods	**0.89 ± 0.01**

In the evaluation of AF detection methods, as reported in table 7, additional artifact coverage for each method is provided in Das (2022). BayesBeat, with an F1 Score of 0.671 at full artifact coverage (without discarding any signals), significantly improved to 0.754 when applying an uncertainty threshold (i.e. keeping only 54% of the signals in the test set). Our method outperformed all others that used the full dataset, achieving an F1 Score of 0.71 with full artifact coverage, indicating a robust and accurate approach for AF detection across diverse signal conditions. Through the AT curve in figure 6(e), we can observe that over half of the signals have more than 90% artifacts. The F1 score can reach up to 90% if signals with over 80% of artifacts are discarded.

**Table 7.** Results compared with SOTA in AF dataset. Only comparisons between Shen *et al* Deapbeat with all data, BayesBeat, and our method use the same data (all 100%). Incomparable results are in gray.

Methods	AF detection (F1 Score)	Artifact Coverage
Shen *et al* 2019 (Voisin *et al* 2019)	0.684	100
Torres-Soto and Ashley (Deepbeat, all data) (Torres-Soto and Ashley 2020)	0.652	100
Torres-Soto and Ashley (Deepbeat, Non-low) (Torres-Soto and Ashley 2020)	0.646	27.67
Torres-Soto and Ashley (Deepbeat, Excellent only) (Torres-Soto and Ashley 2020)	0.58	18.77
BayesBeat (Das 2022)	0.671	100
BayesBeat (uncertainty threshold = 0.05) (Das 2022)	0.754	54
Our methods	0.71	100

In the study of PPG-based SBP estimation tasks, as reported in table 8, the CNN-GRUattn method, which employs a blend of Convolutional Neural Networks and Gated Recurrent Units with attention mechanisms and uses both ECG and PPG signals, achieved a MAE of 4.90. UTransBPNet, also integrating ECG and PPG data, improved upon this accuracy with an MAE of 4.38. In contrast, our method, which solely relies on PPG signals, recorded a worse MAE of 8.6, indicating a lower accuracy in SBP prediction compared to the multi-signal approaches. Even across the AT curve, the MAEs remain in a relatively narrow range, from 8.15 to 8.2. Hence, ECG adds value to SBP estimation. However, PPG is collected passively and can offer continuous monitoring, while ECG requires subjects to remain stationary and thus is not nearly as widely available.

**Table 8.** Results compared with SOTA in SBP dataset. Note that our method uses only PPG data, whereas the others use both PPG and ECG data so the results are not comparable.

Method	SBP (MAE)
CNN-GRU_attn_ (ECG, PPG) (Wang *et al* 2023b)	4.90
UTransBPRNet (ECG, PPG) (Zheng *et al* 2023)	4.38
Our methods (PPG only)	8.6 ± 6.93

## 4. Pre-training and fine-tuning configuration

For all the experiments discussed thus far, we fine-tuned all the parameters for each downstream task (we call this ‘fine-tune All’). We also considered a ‘fine-tune Last’ strategy, where the parameters of the pre-trained encoder are frozen while only the newly initialized classifier layer is tuned according to the labels of target tasks). We also tried a third strategy, where we pre-trained SiamQuality on the downstream datasets and then fine-tuned the classification layer. (Note again that the pre-training does not require labels from downstream tasks, since the goal of placing low- and high-quality signals requires only simulated labels—it is a self-supervised method.)

As reported in table 9, fine-tuning all layers of the model yields the best results across multiple metrics, including MAE in TROIKA, DALIA, WESAD, and BP predictions, as well as the F1 Score for AF detection. In contrast, fine-tuning only the last layer significantly diminishes the model’s accuracy, increasing the MAE by about 25% in most datasets and reducing the F1 Score for AF detection by 14.08%. The hybrid approach of in-domain pre-training followed by fine-tuning the last layer offers some improvement over solely fine-tuning the last layer, but it still falls short of the effectiveness achieved by fine-tuning all layers.

**Table 9.** Results compared with different fine-tuning configurations.

	TROIKA	DALIA	WESAD	BP	AF	RR
Fine Tuning configuration	(MAE)	(MAE)	(MAE)	(MAE)	(F1 Score)	(MAE)
Fine-tune All	**4.6**	**6.8**	**5.8**	**8.6**	**0.71**	**0.89**
Fine-tune Last	5.78	8.55	7.3	10.5	0.61	2.2
In-domain pre-training + Fine-tune Last	4.84	7.15	6.1	9.3	0.64	1.6

## 5. Visualization

We explored the latent space of the SiamQuality model by generating simulated PPG signals with varying heart rates using NeuroKit2 (Makowski 2021). To assess the model’s robustness to noise, we incrementally introduced different levels of drift noise, motion amplitude noise, and powerline noise to the PPG signals, ranging from 0 (clean) to 0.7 (very noisy).

**Figure 7.**
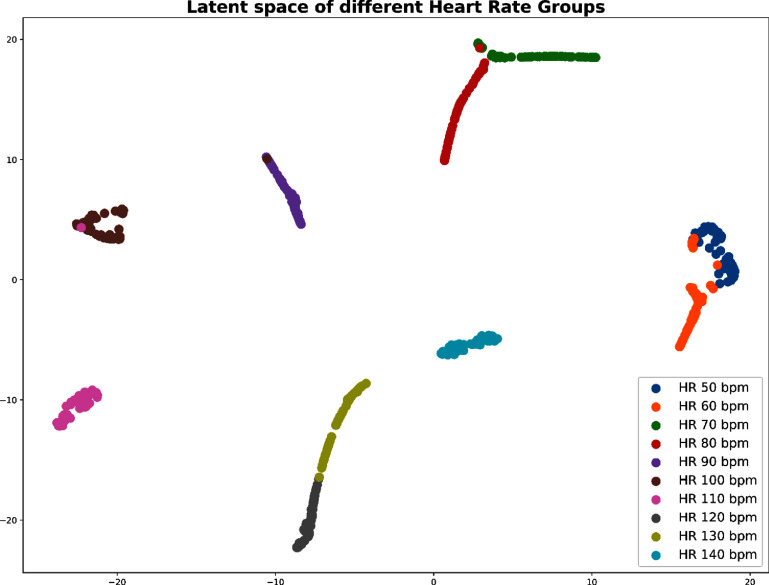
Latent space for simulated PPG with various level of artifacts.

After subjecting these simulated signals to the pre-trained model, we analyzed the resulting latent space. For dimension reduction and visualization, we employed PaCMAP (Wang *et al* 2021), with the findings illustrated in figure 7. Notably, the results reveal that PPG signals with the same heart rate, despite varying noise levels, remain clustered together in the latent space. This observation underscores the efficacy of our proposed quality-pairing mechanism in mitigating the impact of artifacts in PPG signals on downstream physiological measurement tasks.

## 6. Discussion

In this study, we propose SiamQuality, a CNN-based foundation model for PPG signals. SiamQuality utilizes the SimSiam architecture but with a novel pairing mechanism, where we pair one high quality signal with a low quality temporal neighbor. Curriculum learning was applied, where artifact level difference was increased over iterations to make the alignment of PPG representations more difficult. After pre-training on 36 million PPG pairs, the model was then fine-tuned on individual downstream datasets to test four different physiological measurement tasks: estimation of HR, respiratory rate, systolic blood pressure, and detection of AF. Through extensive experiments, we demonstrate the capabilities of SiamQuality in terms of its contrastive learning architecture, scaling ability, and adaptability.

Our approach has three major benefits. First, it avoids selecting negative pairs to train a contrastive learning based foundation model. As PPG signals exhibit strong periodicity, leading to the recurrence of similar patterns within and across patients, conventional augmentation methods may result in choosing inappropriate negative pairs. This hypothesis can be supported by results from table 3. Conventional augmentation for time-series data, such as flipping and adding Gaussian noise, did not perform as well as temporal sampling and was further outperformed by quality-based pairing. This conclusion is in line with the results from Zheng *et al* (2023), which shows that using conventional augmentation methods to construct negative pairs may not be sufficient for time series data, and can lead to performance degradation.

Second, CNNs can serve as an effective backbone for foundation models that are robust to the quality of training data. Through our experiments, CNNs have demonstrated notable efficiency in processing and learning from data with inherent imperfections. This efficiency is attributed to their structural advantage in capturing local patterns through convolutional filters, which inherently makes them less sensitive to global noise and artifacts present in physiological signals.

Third, our previously developed PPG artifact measurement tool (Chen *et al* 2023) plays a crucial role in this study. By providing a quantifiable metric of data quality, it facilitates curriculum learning and the generation of the AT-curve. Curriculum learning enables models to start with pairs less contaminated by noise, thus improving the model’s ability to generalize from cleaner, simpler examples to more complex and noisy data as training progresses. This approach ensures that the model gradually adapts and maintains accuracy across a broader range of conditions, ultimately fostering a deeper and more versatile understanding of the underlying patterns in the data. The AT-curve allows a quantification of the model’s performance when handling different proportions of artifacts; it also reveals the distribution of signals with various artifact levels. As such, the AT-curve is more informative than using a single aggregated performance metric. Points on the AT-curve can be used to estimate expected performance on test sets, even if each test set has a different amount of noise.

As an early-stage investigation into foundation models for physiological data, our work has several limitations.

**Zero-shot learning is not available in our setting**. SiamQuality focuses on aligning the latent space between high and low quality signals, rather than aiming for specific tasks, such as detecting AF or estimating RR. This means that while SiamQuality excels at generalizing across different noise levels of data and ensuring robustness to artifacts and noise, it does not inherently know how to solve new tasks that it was not trained to solve.

**The quality assessment tool is not universal**. We employ a signal quality assessment model that we previously developed specifically for PPG signals to determine their quality as either high or low. This model is tailored exclusively for PPG data. Therefore, if we plan to extend the SiamQuality framework to other types of physiological data, a customized signal quality assessment model will be necessary for each distinct data type.

**Our temporal-quality pairing mechanism is not perfect.** In this study, we introduce a novel pairing mechanism that considers both temporal proximity and signal quality. Specifically, a pair consists of two signals within a 5 min window that have varying levels of signal quality. Our results indicate that this mechanism helps mitigate the effect of low-quality signals, although some concerns persist. One issue is that patients may engage in different activities or transition between different arrhythmias within the 5 min window, representing different physiological states. We recognize this limitation; however, our training dataset was collected in an ICU hospital setting where patients are mostly stationary. Also given the substantial size of our data, instances where the 5 min window overlaps the transition boundary between two different arrhythmias are minimal. In the future, instead of a fixed 5 min window, we can consider dynamic time windows that adjust based on the patient’s activity level or the onset of clinical events. This could help in capturing more physiologically relevant pairings and reduce noise from non-representative signal segments.

**Future work**. All downstream tasks in our study were conducted on publicly accessible PPG benchmarks to ensure reproducibility. While our model achieves or exceeds state-of-the-art results in offline experiments, this does not necessarily imply readiness for real clinical practice. Thus, a crucial area for future work will involve conducting clinical trials and real-world testing to validate and refine our model’s practical utility and reliability in healthcare settings. Additionally, our investigations have predominantly focused on cardiovascular monitoring, where PPG signals are commonly utilized. In the future, it is essential to explore the potential of foundation models to enable PPG in tackling more innovative and less conventional tasks, such as evaluating mental stress levels, monitoring hydration status, and detecting early signs of sepsis. These areas, while challenging, represent significant opportunities to extend the utility of PPG signals beyond traditional cardiovascular applications.

## 7. Reproducibility

The code associated with this project is publicly available at https://github.com/chengding0713/SiamQuality.

## 8. Conclusion

SiamQuality addresses the often-neglected issue of data quality in foundational models. Our focus is on PPG signals—a type of physiological data where quality concerns are particularly pronounced. SiamQuality is based on the SimSiam architecture and includes a unique pairing mechanism that connects a high-quality signal with a temporally adjacent lower-quality signal counterpart. After being pre-trained on a large-scale dataset with 36 million PPG signal pairs, SiamQuality is further fine-tuned on six datasets that span four different downstream tasks. Our results highlight effectiveness of contrastive learning, performance improvements with increasing model size, and versatility in performing different downstream tasks. This study goes beyond merely presenting an efficient model for PPG signals; it demonstrates a general approach to handle low-quality data.

## Data Availability

Due to the nature of the private health data involved, the training dataset is available upon request to the corresponding author. The six downstream datasets are publicly available as reported in table 2.
